# Supplementary material for: Epithelial fusion is mediated by a partial epithelial–mesenchymal transition
Source: Biol Open. 2025 Sep 26;14(9):bio062213. doi: 10.1242/bio.062213 (PMC12505275; doi:10.1242/bio.062213)
Supplement: Supplementary information [file biolopen-14-062213-s1.pdf]

Table S1. SE VS OE

Pathway	Entities found/ Total entities	List of entities	Entities p Value
**Gastrulation**	38/177	TFAP2A;EPHA4;TFAP2B;FOXC2;NOTCH1;WNT3A;DLX5;OSR1;NOG;GATA6;PAX6;GATA4;TBX6;DLL1;PAX2;SOX2;BMP4;SOX1;GBX2;NOTO;MSX1	5.88418203051333E-14
**Specification of the neural plate border**	12/24	TFAP2A;SOX2;BMP4;TFAP2B;GBX2;WNT3A;DLX5;MSX1	3.71760766615381E-09
**Kidney development**	6/25	RET;BMP4;IRX2;FOXC2;OSR1;SIX1;SLIT2;PCDH19;DLL1;PAX2;WNT4	4.81846833544353E-08
**Formation of posterior neural plate**	8/14	TFAP2A;SOX2;BMP4;TFAP2B;GBX2;WNT3A;DLX5;MSX1	5.55479983788132E-07
**WNT ligand biogenesis and trafficking**	9/28	WNT6;WNT10A;WNT2B;WNT3A;WNT7B;WNT7A;WNT9A;WNT4	1.1541409622895E-05
**Extracellular matrix organization**	35/328	COL17A1;COL14A1;NRXN1;NCAN;ITGB3;ELN;ITGB2;CTSV;CTSS;TTR;SPP1;CAPN2;DMD;CTSB;CAST;TGFB2;MUSK;VWF;LAMB2;P3H2;FN1;DCN;BMP4;COL3A1;COL2A1;P4HA1;OPTC;COL9A1;COL8A1;COL9A3;TLL1;COL9A2;FMOD	1.55235048922009E-05
**Degradation of the extracellular matrix**	21/148	CAST;COL17A1;COL14A1;LAMB2;ELN;FN1;CTSV;CTSS;DCN;COL3A1;COL2A1;OPTC;SPP1;COL9A1;CAPN2;COL8A1;COL9A3;TLL1;COL9A2;CTSB	1.60498112887764E-05
**Assembly of collagen fibrils and other multimeric structures**	13/67	COL17A1;COL3A1;COL2A1;COL14A1;COL9A1;COL8A1;COL9A3;TLL1;COL9A2;CTSV;CTSS;CTSB	3.11722598138609E-05
**ECM proteoglycans**	14/79	TGFB2;MUSK;LAMB2;NCAN;ITGB3;FN1;DCN;COL3A1;COL2A1;COL9A1;COL9A3;COL9A2;FMOD	4.13976469858524E-05
**Formation of the anterior neural plate**	7/19	SOX2;SOX1;GBX2;PAX6	4.64329296450128E-05

Biology Open • Supplementary information

Table S2. OV vs OE

Pathway	Entities found/ Total entities	List of entities	Entities p Value
**Gastrulation**	31/177	TFAP2B;NOTCH1;WNT3A;DLX5;PAX3;PAX6;TBX6;DLL1;SOX2;SOX1;FGF8;CDH1;LHX1;ZIC1;NOTO;OTX2;MSX1	6.43880393447205E-10
**Specification of neural plate border**	11/24	SOX2;TFAP2B;WNT3A;DLX5;ZIC1;PAX3;MSX1	2.69769034888512E-08
**Kidney development**	17/75	RET;IRX1;WNT11;SALL1;LHX1;SIX2;SIX1;GFRA1;DLL1	1.46460633176915E-07
**ECM proteoglycans**	17/79	SPARC;MUSK;LAMB2;LUM;NCAN;TNC;HAPLN1;ACAN;VTN;COL3A1;COL1A2;COL5A1;COL6A1;COL9A1;COL9A3;COL9A2	2.99078335874725E-07
**Formation of the posterior neural plate**	8/14	SOX2;SOX1;FGF8;WNT3A;OTX2;TBX6	4.18442687499798E-07
**Extracellular matrix organization**	19/164	COL17A1;SPARC;SDC2;NCAN;ELN;COL12A1;ITGB2;TNC;PLOD1;CTSV;CTSS;FBLN5;HAPLN1;ACAN;VTN;TTR;CDH1;KDR;SPP1;CAPN2;MUSK;MME;LAMB2;LUM;COL22A1;ADAM19;COL3A1;COL1A2;COL5A1;P4HA1;COL6A1;ADAM12;COL9A1;COL8A1;COL9A3;COL9A2	4.20357242902902E-07
**Formation of the ureteric bud**	10/29	RET;WNT11;SALL1;SIX2;SIX1;GFRA1	1.47534758565548E-06
**NCAM1 interactions**	11/44	COL3A1;COL5A1;ST8SIA2;NCAN;COL6A1;COL9A1;GFRA1;COL9A3;CACNA1D;COL9A2;GFRA4	9.27286524665671E-06
**Collagen chain trimerization**	11/44	COL17A1;COL3A1;COL1A2;COL5A1;COL22A1;COL6A1;COL12A1;COL9A1;COL8A1;COL9A3;COL9A2	9.27286524665671E-06
**Integrin cell surface interactions**	15/86	LUM;ITGB2;TNC;VTN;COL3A1;COL1A2;COL5A1;CDH1;COL6A1;KDR;SPP1;COL9A1;COL8A1;COL9A3;COL9A2	1.73014475652877E-05

Biology Open • Supplementary information
